# P01 An unusual case of pregnancy associated osteoporosis presenting with bilateral neck of femur fractures

**DOI:** 10.1093/rap/rkad070.022

**Published:** 2023-09-27

**Authors:** Claire Wood, Visali Sivathavalal, Colin Malcolm, Zhuo Min Chong

**Affiliations:** Department of Rheumatology, University Hospital Wishaw, Glasgow, United Kingdom; Department of Obstetrics and Gynaecology, University Hospital Wishaw, Glasgow, United Kingdom; Department of Obstetrics and Gynaecology, University Hospital Wishaw, Glasgow, United Kingdom; Department of Endocrinology, University Hospital Wishaw, Glasgow, United Kingdom

## Abstract

**Introduction:**

Pregnancy- and lactation-associated osteoporosis (PAO) is a rarely observed condition of skeletal fragility. We present the case of a patient diagnosed with PAO after sustaining bilateral femoral neck fractures one day postpartum. PAO usually presents with vertebral fractures in the third trimester or early postpartum. Femoral fractures are rare, making our case more unusual. PAO is considered a separate entity from transient osteoporosis of the hip (TOH) which is another uncommon cause of hip fracture in pregnancy. Our patient had clinical and imaging features compatible with TOH but with evidence of generalised osteoporosis on DEXA, supporting a diagnosis of PAO.

**Case description:**

Our 35-year-old primigravida has a past medical history of anxiety, depression and schizoaffective disorder. She had been taking high dose sodium valproate for 8 years prior to pregnancy. From the 26th week of her pregnancy she experienced severe and progressive hip and pelvic pain which limited her mobility. She required crutches to mobilize short distances and spent significant periods of time in a wheelchair. Her symptoms were attributed to pubic symphysis dysfunction and managed conservatively. An elective caesarean section was carried out at 37 + 4 weeks. Day one postpartum her leg gave way causing her to trip in the shower and she experienced a sudden and severe worsening of pain.

X-ray revealed a displaced subcapital right femoral neck fracture. MRI confirmed this and showed an additional non-displaced left intracapsular femoral neck insufficiency fracture. She underwent a right total hip replacement and left sided dynamic hip screw fixation.

Blood tests showed normal calcium, phosphate and parathyroid hormone. Vitamin D was 47 ng/ml. TTG antibody was negative. An X-ray of her whole spine excluded any vertebral fractures. Genetic tests for osteogenesis imperfecta were negative. DEXA showed severe osteoporosis in her spine with a T score ranging from −3.7 to −4.1. Her low bone mineral density (BMD) was thought to be due to a combination of relative immobility, long term sodium valproate treatment and PAO.

She was discharged with oral calcium and vitamin D supplementation and advised not to breastfeed. She recovered well and now mobilises independently. She has not sustained any further fractures. Her sodium valproate dose was significantly reduced without worsening of her psychiatric symptoms. Repeat DEXA one year later showed a 10% improvement in BMD at her spine. She now takes high dose vitamin D monotherapy with an aim to keep her vitamin D level above 75 ng/ml.

**Discussion:**

During pregnancy, the foetus has high calcium demands, especially in the third trimester when mineralisation of the fetal skeleton occurs. If the maternal adaptations to meet this demand are insufficient, the risk of maternal skeletal resorption increases. Studies have shown a transient reduction in maternal bone mass during pregnancy and lactation. Women presenting with PAO are often found to have pre-existing conditions or risk factors for reduced BMD. We focused on addressing our patient’s main modifiable risk factor by reducing her sodium valproate dose.

The evidence base for treatment of PAO is lacking and there is no consensus on the best way to manage this condition. Spontaneous improvement in BMD is known to occur in the months following pregnancy and lactation as we observed in our case. Most women are offered calcium and vitamin D supplementation and advised to avoid breast feeding, as our patient was.

Bisphosphonates, denosumab, strontium ranelate and teriparatide have all been tried in individual cases as there is suggestion that these agents may increase BMD more than calcium and vitamin D supplementation alone. However, some experts feel that safety concerns around the use of bisphosphonates during reproductive years outweigh any potential benefits.

PAO usually presents with back pain and/or height loss due to vertebral fractures which are typically multiple. TOH is considered a separate condition which usually presents with acute onset of pelvic, hip or groin pain due to femoral head oedema. The symptoms are severe, progressive and typically affect mobility. In extreme cases fractures may occur. These are usually unilateral but are occasionally bilateral femoral fractures. The gold standard for diagnosis is with MRI which demonstrates oedema of the femoral head and may show an associated joint effusion. This would fit with the clinical history and imaging findings for our patient.

**Key learning points:**

PAO is a rare cause of fragility fractures with an estimated incidence of 4 cases per million pregnancies quoted in the literature. It is important to consider it as a possible cause of hip and pelvic pain in pregnancy. Our patient’s presentation was particularly unusual as there are only a handful of cases in the literature of hip fracture occurring in PAO, bilateral fractures being more uncommon still.

One of the issues for discussion highlighted by this case is the difficulty in knowing how to treat women presenting with fragility fractures due to PAO. Studies have been limited by the rarity of the condition. None of the anti-resorptive or anabolic agents used in osteoporosis are licensed for PAO so the decision to use them needs to be individualized and should consider the potential risks of these treatments in women of child-bearing age. When these agents have been trialed for PAO they have been discontinued prior to any subsequent pregnancies. Cases of shortened gestational age, low birthweight and transient neonatal hypocalcaemia have been observed but no serious adverse maternal or fetal events to date.

Another key discussion point in this case relates to the similarities and differences between the two conditions of PAO and TOH which are both rare causes of fragility fractures. In case reports of TOH, BMD measurement by DEXA has often not been reliably carried out. The diagnosis is commonly made by clinical history and MRI appearances alone. In cases where DEXA has been done it usually demonstrates regional osteoporosis in the affected hip(s) with near normal spinal BMD. However, some cases of TOH report significant generalised osteoporosis in addition which makes distinguishing between these two conditions even more challenging. Thankfully, both conditions generally have a favorable prognosis, as we found with our case.

